# Pathogenic heterozygous TRPM7 variants and hypomagnesemia with developmental delay

**DOI:** 10.1093/ckj/sfae211

**Published:** 2024-07-05

**Authors:** Willem Bosman, Kameryn M Butler, Caitlin A Chang, Mythily Ganapathi, Edwin Guzman, Femke Latta, Wendy K Chung, Felix Claverie-Martin, Jessica M Davis, Joost G J Hoenderop, Jeroen H F de Baaij

**Affiliations:** Department of Medical BioSciences, Radboudumc, Nijmegen, The Netherlands; Greenwood Genetic Center, Greenwood, SC, USA; Department of Medical Genetics, BC Women and Children's Hospital, Vancouver, British Columbia, Canada; Department of Pathology and Cell Biology, Columbia University Irving Medical Center, New York, NY, USA; Department of Pediatrics, Columbia University Irving Medical Center, New York, NY, USA; Department of Medical BioSciences, Radboudumc, Nijmegen, The Netherlands; Department of Pediatrics, Boston Children's Hospital and Harvard Medical School, Boston, MA, USA; Unidad de Investigación, RenalTube Group, Hospital Universitario Nuestra Señora de Candelaria, Santa Cruz de Tenerife, Spain; Greenwood Genetic Center, Greenwood, SC, USA; Department of Medical BioSciences, Radboudumc, Nijmegen, The Netherlands; Department of Medical BioSciences, Radboudumc, Nijmegen, The Netherlands

**Keywords:** autism spectrum disorder, developmental delay, genetics, hypomagnesemia, TRPM7

## Abstract

**Background:**

Heterozygous variants in *Transient receptor potential melastatin type 7* (*TRPM7*), encoding an essential and ubiquitously expressed cation channel, may cause hypomagnesemia, but current evidence is insufficient to draw definite conclusions and it is unclear whether any other phenotypes can occur.

**Methods:**

Individuals with unexplained hypomagnesemia underwent whole-exome sequencing which identified *TRPM7* variants. Pathogenicity of the identified variants was assessed by combining phenotypic, functional and *in silico* analyses.

**Results:**

We report three new heterozygous missense variants in *TRPM7* (p.Met1000Thr, p.Gly1046Arg, p.Leu1081Arg) in individuals with hypomagnesemia. Strikingly, autism spectrum disorder and developmental delay, mainly affecting speech and motor skills, was observed in all three individuals, while two out of three also presented with seizures. The three variants are predicted to be severely damaging by *in silico* prediction tools and structural modeling. Furthermore, these variants result in a clear loss-of-function of TRPM7-mediated magnesium uptake *in vitro*, while not affecting TRPM7 expression or insertion into the plasma membrane.

**Conclusions:**

This study provides additional evidence for the association between heterozygous *TRPM7* variants and hypomagnesemia and adds developmental delay to the phenotypic spectrum of *TRPM7*-related disorders. Considering that the *TRPM7* gene is relatively tolerant to loss-of-function variants, future research should aim to unravel by what mechanisms specific heterozygous *TRPM7* variants can cause disease.

## Graphical Abstract

**Graphical Abstract:**
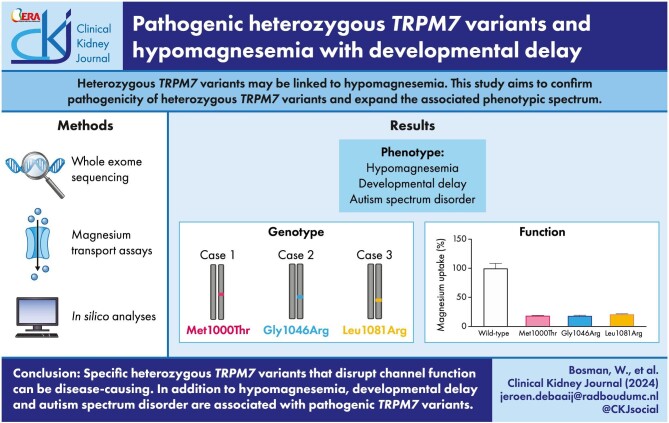

KEY LEARNING POINTS **What was known:**

- Two studies have reported heterozygous variants in *Transient receptor potential melastatin type 7* (*TRPM7*) in three families with hypomagnesemia.
- Considering the low number of cases, current evidence is insufficient to conclude that *TRPM7* variants can indeed be causative for hypomagnesemia.
- Beyond hypomagnesemia, the exact nature of other phenotypes associated with potentially pathogenic *TRPM7* variants is incompletely described.

**This study adds:**

- With the addition of three independent cases, we confirm that heterozygous TRPM7 variants can be disease-causing and lead to hypomagnesemia.
- In addition to hypomagnesemia, TRPM7 variants may also be associated with global developmental delay and autism spectrum disorder.

**Potential impact:**

- TRPM7 can be added to gene panels in the diagnosis of individuals with unsolved hypomagnesemia, developmental delay and/or autism spectrum disorder.

## INTRODUCTION

Magnesium (Mg^2+^) is implicated in many processes in health and disease and its homeostasis has to be maintained tightly by intestinal absorption, storage in bone and soft tissues, and renal reabsorption [1]. Transient receptor potential melastatin type 7 (TRPM7) is a divalent cation channel responsible for the influx of ions such as Mg^2+^, calcium (Ca^2+^) and zinc [2]. Although the channel is ubiquitously expressed, within the kidney TRPM7 expression is significantly higher in the distal convoluted tubule (DCT), the only segment of the nephron where transcellular Mg^2+^ reabsorption occurs [3–5]. Recently, two independent groups identified heterozygous variants in the *TRPM7* gene in three families with hypomagnesemia (serum Mg^2+^ <0.7 mmol/L) [6, 7]. In addition, these studies report secondary hypocalcemia, seizures and hemiplegic migraine attacks as potential consequences of the *TRPM7* variants. These phenotypes are reminiscent of hypomagnesemia with secondary hypocalcemia (HSH, MIM #602 014) caused by bi-allelic variants in *TRPM6* [8, 9]. The hypocalcemia can be explained by inadequately low parathyroid hormone (PTH) levels, caused by a hypomagnesemia-induced reduction in PTH production and secretion [10]. Sporadically, individuals with HSH also present with neurological symptoms such as intellectual disability and psychomotor delays, both observed in 3 out of 88 reported cases [11]. Since TRPM6 and TRPM7 are thought to function as heterotetramers to facilitate Mg^2+^ (re)absorption in colon and DCT and thereby maintain sufficient Mg^2+^ levels in the body [12], it is not surprising that *TRPM7* variants could cause a similar phenotype. However, TRPM7 is more broadly expressed and has various TRPM6-independent functions, suggesting that phenotypes associated with *TRPM7* variants could be more extensive.

Considering the low number of reported cases, the current evidence is insufficient to unequivocally conclude that heterozygous *TRPM7* variants cause hypomagnesemia and it is unclear which other phenotypes can occur. Therefore, the aim of this study is to characterize additional *TRPM7* variants in order to provide additional evidence for their pathogenicity and enhance understanding of the associated phenotypic spectrum. This led to the identification of three novel variants that were tested for effects on protein expression, structure and function.

## MATERIALS AND METHODS

### Study participants

Individuals were included when a novel *TRPM7* variant that was suspected but unconfirmed to be causative for their phenotypes was identified. GeneMatcher was employed to find and connect multiple cases [13]. Variant identification was part of routine genetic diagnostics without the need for additional procedures. Written informed consent was obtained for the genetic analysis and the publication of anonymized data.

### Genetic analysis

Variants were identified by whole-exome sequencing (WES) at the respective institutes: Columbia University Irving Medical Center, NY, USA by reanalysis of WES data in 2023. The clinical WES was previously done at GeneDx (Stamford, CT, USA) in 2013 and was non-diagnostic (Case 1); Greenwood Genetic Center, SC, USA using the SureSelectXT Clinical Research Exome kit (Agilent) and the NovaSeq 6000 System (Illumina) with 150-bp paired-end reads (Case 2); and University of British Columbia, Canada in collaboration with GeneDx for sequencing in 2022 (Case 3). Reads were aligned to human genome build GRCh37/hg19. Only canonical splice site or non-synonymous coding variants with five or more reads, variation in ≥20% of reads and minor allele frequencies <0.1% were included. Allele frequencies of the newly identified *TRPM7* variants were checked in the GnomAD v2.1.1 [14] and GME Variome [15] databases in September 2023. For *in silico* prediction of pathogenicity, REVEL [16] and BayesDel v1 [17] scores were used with cut-off values of ≥0.644 and ≥0.13 based on the analysis by Pejaver *et al.* [18].

### DNA constructs

The pTracer-CMV2 construct encoding mouse TRPM7 (NM_021450.2; 97% sequence similarity with human TRPM7) with a carboxy-terminal HA-tag was a kind gift from Dr David E. Clapham. The variants were introduced using the Q5 site-directed mutagenesis kit (New England BioLabs; E0554) following the manufacturer's instructions.

### Cell culture and transfection

Human Embryonic Kidney 293 (HEK293) cells were maintained in Dulbecco's Modified Eagle’s Medium (Gibco, 42430-035) supplemented with 10% (v/v) fetal bovine serum, 1 mmol/L sodium pyruvate and 0.1 mmol/L non-essential amino acids (Westburg, CA NEAA-B) at 37°C, 5% (v/v) CO_2_. Lipofectamine 2000 (Invitrogen, 11668-019) was used for transfection at a 1:2 DNA to Lipofectamine ratio according to the manufacturer's instructions.

### ^25^Mg^2+^ uptake assay

HEK293 cells were seeded in 12-well plates (three wells per condition) coated with poly-l-lysine (Sigma-Aldrich, P2636). Six hours later, cells were transfected with empty vector, wild-type (WT) or variant *Trpm7*. Two days after transfection, cells were washed once with uptake buffer (125 mmol/L NaCl, 5 mmol/L KCl, 0.5 mmol/L CaCl_2_, 0.5 mmol/L Na_2_HPO_4_, 0.5 mmol/L Na_2_SO_4_, 15 mmol/L HEPES, set to pH 7.5 with NaOH) without Mg^2+^ and then incubated for 15 min in uptake buffer containing 1 mmol/L ^25^MgO (CortecNet, M-Mg25). Cells were then washed three times with ice-cold phosphate-buffered saline (PBS), lysed in HNO_3_ and analyzed with inductively coupled plasma mass spectrometry.

### Cell surface biotinylation

HEK293 cells were seeded in poly-l-lysine-coated six-well plates (two wells per condition) and transfected with empty vector, WT or mutant *Trpm7*. Two days later, cells were transferred to a cold room (4°C) and washed twice with PBS-CM (PBS with 0.5 mmol/L CaCl_2_ and 1 mmol/L MgCl_2_, pH 8.0). PBS-CM containing 0.5 mg/mL Sulfo-NHS-LC-LC-Biotin (Thermo Scientific, 21 338) was then added to the cells for 30 min. For cells transfected with WT, a negative control without biotin was included to confirm specificity of the assay. After washing twice in PBS-CM with 0.1% bovine serum albumin and twice in PBS, cells were lyzed in lysis buffer [1% (v/v) Triton X-100, 50 mmol/L Tris-HCl pH 7.5, 1 mmol/L EDTA, 1 mmol/L EGTA, 10 mmol/L sodium glycerophosphate, 50 mmol/L NaF, 10 mmol/L sodium pyrophosphate, 270 mmol/L sucrose, 150 mmol/L NaCl] containing freshly added protease and phosphatase inhibitors (1 mmol/L phenylmethylsulfonyl fluoride, 5 µg/mL leupeptin hemisulfate, 1 µg/mL pepstatin A, 1 µg/mL aprotonin, 1 mmol/L sodium orthovanadate). Equal amounts of proteins were added to 20 µL NeutrAvidin Agarose Resin (Thermo Scientific, 29 201) and input samples were prepared to check expression. Samples were incubated with the Resin overnight while rotating. After washing three times with lysis buffer, samples were eluted with 40 µL of 2× Laemmli/dithiothreitol buffer at 37°C for 30 min.

### SDS-PAGE and western blotting

Biotinylation samples were loaded on 8% (v/v) SDS-PAGE gels and transferred to polyvinylidene difluoride membranes. After blocking with 5% (w/v) milk in TBS-Tween, membranes were incubated overnight with primary antibody targeting HA (Cell Signaling Technology, 2367) diluted 1:5000 or β-actin (Sigma-Aldrich; A5441) diluted 1:10 000 in 1% (w/v) milk. Blots were then incubated for 1 h with peroxidase-conjugated secondary antibody (Jackson ImmunoResearch, 515-035-003) diluted 1:10 000 and visualized with PICO chemiluminescent substrate (ThermoFisher, 34 580).

### *In silico* modeling

The following reference sequences were used for alignment of the TRPM7 amino acid sequence of different species: NP_060142.3 (*Homo sapiens*), NP_446157.2 (*Rattus norvegicus*), NP_067425.2 (*Mus musculus*), NP_001384973.1 (*Gallus gallus*), NP_001025232.1 (*Danio rerio*). The variants were modeled in the structure of closed apo state mouse TRPM7 reconstituted in lipid nanodiscs solved by cryogenic electron microscopy (Protein Data Bank, 8SI2) [19]. Considering the heterozygosity, the variants should affect TRPM7 function even if not all subunits of the tetramer are affected, so they were modeled in only one subunit. Analysis and visualization were performed in YASARA [20].

### Statistical analysis

Welch's ANOVA with Dunnett's T3 multiple comparisons test was used to compare ^25^Mg^2+^ uptake capacity and cell surface expression between WT and variants. A *P*-value of <.05 was considered statistically significant.

## RESULTS

### Hypomagnesemia and developmental delay in individuals with novel *TRPM7* variants

To provide an overview of phenotypes associated with heterozygous *TRPM7* variants, characteristics of three individuals with newly identified variants are summarized together with the five cases from previous studies (Table 1) [6, 7]. The three novel cases are described in more detail below.

**Table 1:** Characteristics of affected individuals.

**Individual**	**Case 1**	**Case 2**	**Case 3**	**Lei et al. 2022**	**F1 II.2 Vargas-poussou et al. 2023**	**F1 II.3 Vargas-Poussou et al. 2023**	**F1 III.3 Vargas-Poussou et al. 2023**	**F2 II.1 Vargas-Poussou et al. 2023**
*TRPM7* Variant	p.Met1000Thr	p.Gly1046Arg	p.Leu1081Arg	p.Met1000Val	c.3+1G>C			p.Gly1046Asp
Sex	F	M	M	M	F	M	M	M
Hypomagnesemia (mmol/L)	Y (0.37)	Y (0.45-0.62)	Y (0.47-0.50)	Y (0.36-0.56)	Y (0.49)	Y (0.25)	Y (0.51)	Y (0.61)
Hypocalcemia	N	NA	N	NA	Y	Y	N	Y
Hypoparathyroidism	N	NA	N	N	N	Y	N	N
Seizures	Y	Y	N	N	N	Y	N	Y
Developmental delay	Y	Y	Y	N	N	N	N	Y
Motor skill defects	Y	Y	Y	Y	N	N	N	Y
Speech abnormalities	Y	Y	Y	Y	N	N	N	Y
Other	ASD, ADHD	ASD, eczema, OSA, enamel hypoplasia/ amelogenesis imperfecta	ASD, dysmorphic features, *MT-CO1* variant	Hemiplegic attacks	Hyper-cholesterolemia, Hashimoto thyroiditis, muscle cramps	Dyslipidemia, CKD, Graves'disease	Asthma, paresthesia	ASD, Nystagmus

ADHD, attention deficit hyperactivity disorder; CKD, chronic kidney disease; F, female; M, male; N, no; NA, not assessed; OSA: obstructive sleep apnea; Y, yes

Case 1 is a 14-year-old girl with global developmental delay, seizures, autism spectrum disorder (ASD) and attention deficit hyperactivity disorder. Her developmental delay is characterized by speech impairment (first words at 32 months, echolalic speech), motor skill disorders (dyspraxia) and borderline normal intellectual functioning. She also has torticollis and severe myopia, and shows rapid weight gain. Brain magnetic resonance imaging (MRI) showed no abnormalities (data not shown). Serum analysis revealed hypomagnesemia (0.37 mmol/L) but normal Ca^2+^ (2.36 mmol/L) and PTH levels (3.8 pmol/L). A *de novo* missense variant was identified in *TRPM7* (NM_017672.6: c.2999T>C, p.Met1000Thr) after WES trio analysis, which was the most likely candidate out of 12 identified variants (Supplementary data, Table S1).

Case 2 is a 2-year-old boy with global developmental delay (absent speech and motor skill disorder), seizures, ASD and eczema. In addition, he was recently diagnosed with obstructive sleep apnea and enamel hypoplasia. Brain MRI did not show abnormalities (data not shown). Out of the candidate variants identified after WES trio analysis (Supplementary data, Table S2), only a *de novo* variant (c.3136G>C, p.Gly1046Arg) in *TRPM7* was not inherited from the healthy parents. Subsequently, serum Mg^2+^ was measured at 0.58 mmol/L. He is treated with Topamax and Onfi for the seizures and magnesium supplements were started, resulting in some improvements in sleep and attention, but Mg^2+^ levels remained low (0.45–0.62 mmol/L).

Case 3 is a 4-year-old boy with ASD and developmental delay, characterized by speech delay (first words at 18 months) and motor skill disorders (dyspraxia, axial hypotonia, wide-based gait with toe-walking). There are some dysmorphic features in the face and hands (upslanting palpebral fissure, bulbous nasal tip, low-set ears, persistent fetal pads). MRI was unremarkable apart from an incidental suspicion of hemangioma at the T3 vertebral body (data not shown) and he does not have a history of seizures. He also presented with hypomagnesemia (0.47–0.50 mmol/L) but normal serum Ca^2+^ (2.47 mmol/L) and PTH (6.4 pmol/L). The p.Leu1081Arg (c.3242T>G) variant was identified in *TRPM7* after singleton WES analysis. Targeted Sanger sequencing in the parents confirmed a *de novo* origin. In addition, a maternally inherited variant was found in the mitochondrial gene encoding the cytochrome c oxidase I (MT-CO1) subunit of Complex IV of the electron transport chain (m.7023G>A; YP_003024028.1: p.Val374Met). This variant was considered not to be the disease cause based on its presence in the healthy mother and a very low blood heteroplasmy level (2%).

None of the variants is present in the GnomAD and GME Variome databases, while all three are predicted to be pathogenic by the *in silico* tools (Table 2).

**Table 2:** Variant characterization.

cDNA (c.)	Protein (p.)	GnomAD	GME Variome	REVEL score (cut-off ≥0.644)	BayesDel score (cut-off ≥0.13)
2999T>C	Met1000Thr	Not found	Not found	0.834	0.18069
3136G>C	Gly1046Arg	Not found	Not found	0.975	0.606113
3242T>G	Leu1081Arg	Not found	Not found	0.972	0.599946

### p.Met1000Thr, p.Gly1046Arg and p.Leu1081Arg severely affect TRPM7 function while membrane expression is maintained

**Figure 1:**
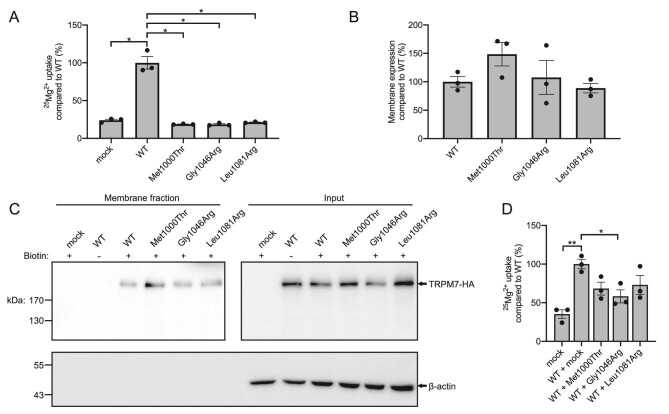
Effects of TRPM7-p.Met1000Thr, p.Gly1046Arg and p.Leu1081Arg on Mg^2+^ uptake and cell membrane expression. (**A**) ^25^Mg^2+^ uptake after 15 min in cells transfected with mock, WT or variant TRPM7. (**B**) Quantification of cell surface biotinylation assay for HEK293 cells transfected with WT or variant TRPM7. (**C**) Representative western blot of cell surface biotinylation assay. (**D**) ^25^Mg^2+^ uptake after 15 min in cells co-transfected with WT TRPM7 with either mock or variant. Data points represent three independent experiments. Data are presented as mean ± standard error of the mean. *P < .05; **P < .01.

To obtain functional evidence for the pathogenicity of the newly identified variants, we performed an uptake assay using the stable isotope ^25^Mg^2+^. Compared with cells transfected with WT TRPM7, p.Met1000Thr (18.7%, *P *= .025), p.Gly1046Arg (18.4%, *P *= .025) and p.Leu1081Arg (21.1%, *P *= .027) demonstrated severely reduced uptake capacity (Fig. 1A). These levels were comparable to cells transfected with the empty vector (23.9% compared with WT), indicating that all three variants result in a complete loss-of-function. We also examined whether variant TRPM7 proteins are present at the plasma membrane by cell surface biotinylation. Overall protein expression was comparable to WT (Supplementary data, Fig. S1A) and all variants were present at the cell surface. The relative membrane expression of all three variants was not significantly different compared with WT (149%, 108% and 89% for p.Met1000Thr, p.Gly1046Arg and p.Leu1081Arg, respectively) (Fig. 1B and C). To assess potential dominant-negative effects, we co-expressed WT TRPM7 with either empty vector or the variants and measured ^25^Mg^2+^ uptake. Total TRPM7 expression was ∼2-fold higher in the presence of the variants compared with the empty vector, indicating WT TRPM7 expression was similar in all conditions (Supplementary data, Fig. S1B and C). When the p.Gly1046Arg variant was co-expressed, uptake capacity was significantly reduced compared with WT alone (58.3%, *P *= .049) (Fig. 1D). Although co-transfection with the p.Met1000Thr and p.Leu1081Arg variants also seemed to affect Mg^2+^ uptake, no significant reductions in Mg^2+^ uptake by WT TRPM7 were observed (68.3 and 73.0%, *P *= .11 and .36, respectively).

**Figure 2:**
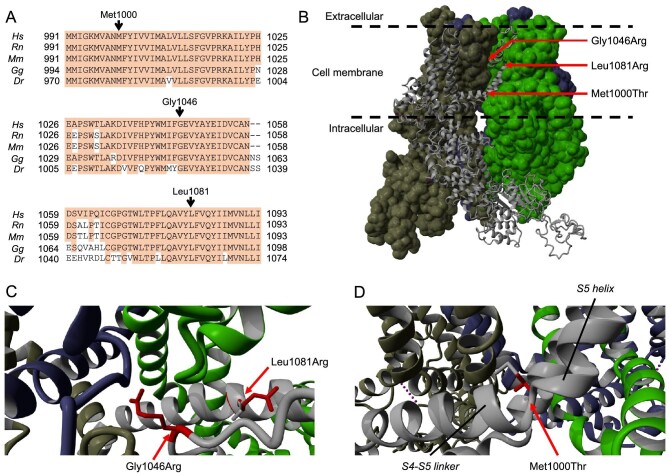
Visualization of TRPM7-p.Met1000Thr, p.Gly1046Arg and p.Leu1081Arg in the TRPM7 protein structure. (**A**) Alignment of the TRPM7 amino acid sequence of different species for the affected residues and surrounding region. Dr, *Danio rerio*; Gg, *Gallus gallus*; Hs, *Homo sapiens*; Mm, *Mus musculus*; Rn, *Rattus norvegicus*. (**B**) Position of the variants in the complete apo state TRPM7 tetramer structure. One subunit (gray) is shown as ribbon diagram, while the other three are shown as Cryo-EM density maps. (**C**) Zoom-in of the Gly1046 vicinity, showing the Gly1046Arg substitution blocking the pore and the Leu1081Arg substitution affecting the S6 helix just below the pore-forming region. (**D**) Zoom-in of the Met1000 vicinity, showing the position of this residue in the bend between the S4-S5 linker and the S5 helix. WT residues are shown in gray, variant residues in red. Unaffected subunits of the tetramer are shown in light green, dark green and blue.

To support our functional findings, we assessed conservation of the affected residues in different species and modeled the amino acid substitutions in the Cryo-EM structure of the TRPM7 tetramer (Fig. 2A and B). All three residues are highly conserved among vertebrates. The Gly1046 residue is located in the pore-forming domain, where the small glycine residue allows passage of cations through the pore. In the p.Gly1046Arg variant, the pore is blocked by the introduction of the large arginine side chain (Fig. 2C). The Leu1081 residue is part of the S6 transmembrane helix, which partially lines the pore. Residues 1078–1083 form a hydrophobic region of this helix that is present right below the pore, in close proximity to Gly1046 (Fig. 2C). In the p.Leu1081Arg variant a large hydrophilic side chain is introduced at the center of this hydrophobic region. Lastly, the Met1000 residue is at the start of the S5 transmembrane helix, where the S5 helix bends in relation to the S4-S5 linker (Fig. 2B and D). The S5 helix is involved in the channel's transition from a closed to an open conformation by moving relative to the S4-S5 linker and away from the pore center [19]. The interaction of the methionine side chain at this bend with other residues is expected to be lost when replaced by the smaller threonine residue, similar to what was observed for p.Met1000Val [6].

## DISCUSSION

In this study, we provide evidence that heterozygous pathogenic *TRPM7* variants are causative for hypomagnesemia and can also be associated with developmental delay and ASD. This is based on the identification of three *de novo* variants (p.Met1000Thr, p.Gly1046Arg and p.Leu1081Arg) in individuals with a similar combination of low serum Mg^2+^ levels and developmental delay with ASD. Two of the three variants affect the same residues, Met1000 and Gly1046, as two recently reported missense variants [6, 7]. In addition, all three variants severely affect the Mg^2+^ uptake capacity of TRPM7 in HEK293 cells and are predicted to be damaging by various *in silico* methods. This work demonstrates that heterozygous *TRPM7* variants at specific positions can be disease-causing and that the resulting phenotypes may be broader than previously described.

Our results combined with the cases from earlier work have now led to the identification of six heterozygous *TRPM7* variants causative for hypomagnesemia—five missense variants and one splice variant. Our data indicate that individuals with *TRPM7* missense variants can also present with global developmental delay, especially affecting motor skills and speech. Although global developmental delay was not reported in the two previous studies, Lei *et al.* do report difficulties with ordered movement and slurred speech in a child with variant p.Met1000Val, while case F2-II.1 described by Vargas-Poussou *et al.* (p.Gly1046Asp) was too young at the time of examination to draw conclusions concerning developmental delay [6, 7]. Indeed, clinical follow-up of this child revealed delays in maturation and speech. This same study also described a family that carries a splice variant (c.3+1G>C), which leads to loss of expression of the affected allele as it causes defective splicing and a premature stop codon in exon 2. Developmental delay seems to be absent and ages of diagnosis were much higher in this family, indicative of a less severe phenotype compared with the missense variants. However, with only one splice variant described so far, it is too early to conclude whether a less severe phenotype is a specific characteristic of such a variant or if it is simply part of the variable phenotypic spectrum of *TRPM7*-related disorders.

Since TRPM7 can form functional heteromeric channels with TRPM6, it is not surprising that the phenotypes of individuals with *TRPM7* variants show similarities to HSH. In both disorders, hypomagnesemia is consistently observed as well as seizures in the majority of cases. Considering both TRPM proteins are co-expressed highly in the colon and DCT, it is likely that in both cases the underlying mechanism of the hypomagnesemia is intestinal malabsorption and renal wasting caused by decreased functioning of TRPM6/TRPM7 heterotetramers. Indeed, a combined reduction in intestinal and renal (re)absorption of Mg^2+^ has been observed in both disorders [7, 8]. Furthermore, in line with the cases from this study, HSH is sometimes also associated with neurodevelopmental disorders, especially when hypomagnesemia is detected and treated late [10, 21, 22]. However, there also seem to be some distinct differences between the disorders. Based on the small cohort described so far, neurodevelopmental delay seems to be more prevalent in *TRPM7*-related disorders compared with HSH, also indicated by the presence of ASD in all three cases described here. Clinical follow-up of the patient carrying variant p.Gly1046Asp has shown ASD as well. A broader and more severe phenotype could be a result of the more ubiquitous expression and general function of *TRPM7* compared with *TRPM6* [23, 24]. Indeed, variants in *TRPM7* have been associated with increased susceptibility to a wide range of diseases, including neurodegenerative disorders [25], colorectal cancer [26], breast cancer [27] and thrombocytopenia [28]. In line with the widespread role of TRPM7 and the heterozygous state of *TRPM7* variants described thus far, global deletion of TRPM7 is not compatible with life in cell and animal models [24, 29]. Considering this broad and essential function of TRPM7, identification of a larger cohort of individuals with pathogenic *TRPM7* variants is required to obtain a more accurate and complete understanding of the associated phenotypes. It is therefore essential that *TRPM7* variants are considered in the diagnosis of individuals with unsolved hypomagnesemia with or without developmental delay and ASD.

It is important to note that GnomAD reports a relatively high number of predicted loss-of-function variants in *TRPM7* in the healthy population, resulting in a probability of being loss-of-function intolerant (pLI) of 0. If haploinsufficiency is assumed as the disease mechanism, these loss-of-function variants would be expected to be disease-causing. This indicates that only specific variants are pathogenic, which is supported by the fact that two of the three novel variants (p.Met1000Thr and p.Gly1046Arg) affect the same residue as previously reported cases. Our data suggest an equally important role for the Leu1081 residue. Indeed, similar to Met1000 and Gly1046, there are no missense variants reported at position Leu1081 in GnomAD or GME Variome. Structurally, the importance of these residues can be understood, as they are predicted to cause significant alterations either directly in the pore region (p.Gly1046Arg, p.Gly1046Asp and p.Leu1081Arg) or in the gating machinery (p.Met1000Thr and p.Met1000Val). In addition, there are other examples of autosomal dominant disorders caused by specific variants in genes that are otherwise tolerant to loss-of-function. Heterozygous variants in the calcium-sensing receptor gene *CASR* (pLI = 0.05) can cause hypocalciuric hypercalcemia [30] while autosomal-dominant tubulointerstitial kidney disease can be caused by variants in *UMOD* (pLI = 0), *MUC1* (pLI = 0.02) or *REN* (pLI = 0) [31]. These observations might be explained by toxic accumulation of mutant protein such as in the case of *MUC1*, in which all pathogenic variants are frameshift variants that result in the same aberrant protein [32], although our data do not point towards accumulation of TRPM7 variants. Alternatively, variants could have specific effects on protein–protein interactions. In addition to TRPM6, TRPM7 interacts with Cyclin and CBS-domain Divalent Metal Cation Transport Mediator proteins (CNNMs), Phosphatases of Regenerating Liver proteins (PRLs) and ADP Ribosylation Factor Like GTPase 15 (ARL15), all of which are implicated in Mg^2+^ homeostasis [33, 34]. Lastly, the most likely explanation for variants affecting the pore region is a dominant-negative effect. The finding that TRPM7-mediated ^25^Mg^2+^ uptake is reduced when the p.Gly1046Arg variant is co-expressed indeed indicates that this variant interferes with WT TRPM7 functioning. However, although trends were observed, these findings could not be replicated for p.Met1000Thr, p.Leu1081Arg or p.Gly1046Asp [7]. This could be explained by less severe effects or the number of subunits of the tetramer that has to be affected. Of note, the aforementioned disease mechanisms do not explain the pathogenicity of the c.3+1G>C variant, as this variant results in loss of TRPM7 expression rather than expression of a non-functional or toxic protein. It is possible that the disease mechanism of the splice variant differs, considering the less severe phenotype of the family carrying this variant. Evidently, although a dominant-negative effect seems likely for the p.Gly1046Arg variant, understanding why only certain variants in *TRPM7* are disease-causing while other loss-of-function variants are not is still an important question for future research.

In conclusion, we have identified three novel pathogenic *TRPM7* missense variants in individuals with hypomagnesemia and developmental delay. These variants affect essential residues in the TRPM7 protein and completely abolish Mg^2+^ uptake capacity. By screening the *TRPM7* gene in individuals with hypomagnesemia, developmental delay and/or ASD the diagnosis of these disorders can be improved and the phenotypic spectrum of TRPM7-related disorders can be expanded.

## Supplementary Material

sfae211_Supplemental_Files

## Data Availability

The variants described in this study have been submitted to ClinVar (p.Met1000Thr: SCV004175909; p.Gly1046Arg: SCV003799049; p.Leu1081Arg: SCV004175910). All relevant data are present within the manuscript. Additional data can be requested from the corresponding author.
