# Supplementary material for: Pathogenic heterozygous TRPM7 variants and hypomagnesemia with developmental delay
Source: Clin Kidney J. 2024 Jul 5;17(8):sfae211. doi: 10.1093/ckj/sfae211 (PMC11295107; doi:10.1093/ckj/sfae211)
Supplement: sfae211_Supplemental_Files [file sfae211_supplemental_files.zip › Supplementary tables.docx]

**Supplementary table 1. Rare variants identified in case 1.**

| *De novo* | | | | | | |
| --- | --- | --- | --- | --- | --- | --- |
| **Chr** | **Position** | **Ref** | **Var** | **Gene** | **Effect** | **Freq** |
| 2 | 27337939 | G | C | GTF3C2 | Missense | 6.9x10^-7^ |
| 4 | 52028925 | C | CA | SGCB | Splice region | 6.2x10^-5^ |
| 10 | 22328010 | C | T | BMI1 | Missense | 6.9x10^-7^ |
| 15 | 50599286 | A | G | TRPM7 | Missense | NR |
| 22 | 21628749 | C | T | YDJC | Missense | 2.2x10^-6^ |
| **Homozygous** | | | | | | |
| **Chr** | **Position** | **Ref** | **Var** | **Gene** | **Effect** | **Freq** |
| 1 | 158547681 | G | A | OR6Y1 | Missense | 9.2x10^-3^ |
| **Compound heterozygous** | | | | | | |
| **Chr** | **Position** | **Ref** | **Var** | **Gene** | **Effect** | **Freq** |
| 2 | 178560481 | G | T | TTN | Missense | 1.8x10^-4^ |
| 2 | 178732482 | C | T | TTN | Missense | 2.5x10^-6^ |
| 2 | 178749359 | G | C | TTN | Missense | 1.9x10^-5^ |
| 2 | 178764173 | C | T | TTN | Splice region | 2.1x10^-6^ |
| 2 | 140315105 | T | A | LRP1B | Splice region | 5.6x10^-6^ |
| 2 | 140371265 | G | A | LRP1B | Missense | 1.3x10^-5^ |

Only rare *de novo,* homozygous or compound heterozygous variants in coding or splice regions were included. Synonymous variants were excluded. Chr, chromosome; Freq, frequency in GnomAD; Ref, reference; Var, variant.

**Supplementary table 2. Rare variants identified in case 2.**

| Chr | Position | Ref | Var | Gene | Effect | Inheritance | Freq |
| --- | --- | --- | --- | --- | --- | --- | --- |
| 8 | 27463139 | G | A | CHRNA2 | Missense | Paternal | 1.2x10^-6^ |
| 12 | 51721597 | A | G | SCN8A | Missense | Paternal | NR |
| 15 | 50599149 | C | G | TRPM7 | Missense | *De novo* | NR |
| 19 | 12676016 | C | T | DHPS | Splice region | Maternal | 3.4x10^-4^ |

Only rare *de novo* variants or rare variants from an epilepsy panel in coding or splice regions were included. Synonymous variants were excluded. Chr, chromosome; Freq, frequency in GnomAD; Ref, reference; Var, variant.
