# Supplementary figures and images for: Pathogenic heterozygous TRPM7 variants and hypomagnesemia with developmental delay
Source: Clin Kidney J. 2024 Jul 5;17(8):sfae211. doi: 10.1093/ckj/sfae211 (PMC11295107; doi:10.1093/ckj/sfae211)

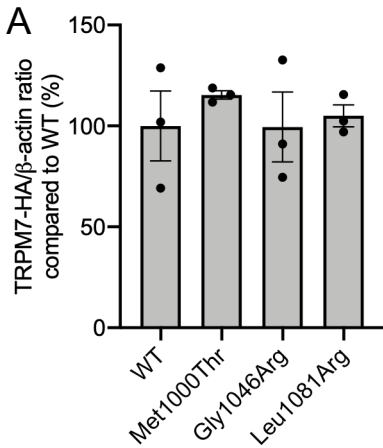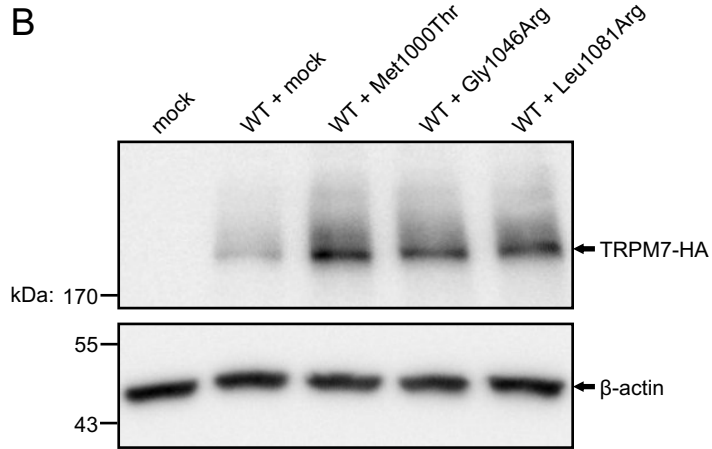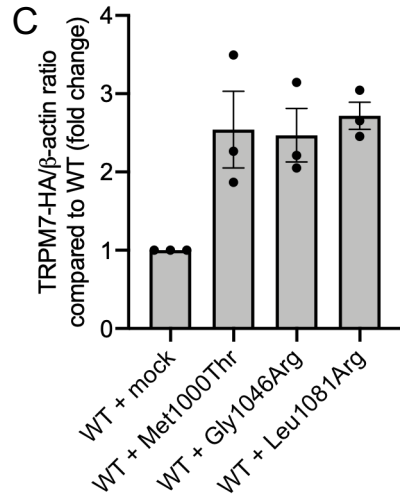

Supplement: sfae211_Supplemental_Files [file sfae211_supplemental_files.zip › FigureS1.pdf]
